# Machine learning models, trusted research environments and UK health data: ensuring a safe and beneficial future for AI development in healthcare

**DOI:** 10.1136/jme-2022-108696

**Published:** 2023-03-30

**Authors:** Charalampia (Xaroula) Kerasidou, Maeve Malone, Angela Daly, Francesco Tava

**Affiliations:** 1 School of Medicine, University of Dundee, Dundee, UK; 2 Dundee Law School, School of Humanities Social Sciences and Law, University of Dundee, Dundee, UK; 3 Leverhulme Research Centre for Forensic Science, School of Science and Engineering, University of Dundee, Dundee, UK; 4 School of Social Sciences, UWE Bristol, Bristol, UK

**Keywords:** ethics, ethics- research, information technology, policy, ethics- medical

## Abstract

Digitalisation of health and the use of health data in artificial intelligence, and machine learning (ML), including for applications that will then in turn be used in healthcare are major themes permeating current UK and other countries’ healthcare systems and policies. Obtaining rich and representative data is key for robust ML development, and UK health data sets are particularly attractive sources for this. However, ensuring that such research and development is in the public interest, produces public benefit and preserves privacy are key challenges. Trusted research environments (TREs) are positioned as a way of balancing the diverging interests in healthcare data research with privacy and public benefit. Using TRE data to train ML models presents various challenges to the balance previously struck between these societal interests, which have hitherto not been discussed in the literature. These challenges include the possibility of personal data being disclosed in ML models, the dynamic nature of ML models and how public benefit may be (re)conceived in this context. For ML research to be facilitated using UK health data, TREs and others involved in the UK health data policy ecosystem need to be aware of these issues and work to address them in order to continue to ensure a ‘safe’ health and care data environment that truly serves the public.

## Introduction

There is a broad structural shift taking place in the UK and beyond,^i^ which ushers in the increasing digitisation of the health and care sector. This is a shift that is balancing between two diverging yet interconnected developments: an increasing appetite for data-driven and machine learning (ML) healthcare technologies, supported by an innovation-driven research, technology and policy sector; and increasing awareness of the importance of legal and ethical safeguards guiding such innovations to ensure that legal rights and obligations, such as confidentiality and privacy, are protected and upheld along with more ethical approaches including the public’s continued collaboration in such endeavours.^1 2^ In the UK, trusted research environments (TREs) sit at the junction of these developments, attempting to balance differing interests between the public, research and rights.

TREs, also known as ‘data enclaves’, ‘research data centre/centres’ or ‘safe havens’, are physical or virtual analytical environments which can hold various data sets (such as population, census, or healthcare data, etc). Subject to monitoring and access controls, a TRE user can be allowed to work with these data but is prevented from releasing their analysis without permission. The aim of TREs is to provide a secure location for researchers to analyse data, especially personal data, enabling collaborative and transparent research while protecting data confidentiality and privacy.

While TREs have received relatively little attention in academic literature and debate, especially from ethical perspectives,^3 4^ they are not new.^ii^ Some have been in operation for almost 20 years now. Yet, they have recently come to the limelight as a key service for the UK's National Health Service (NHS) data which can engender public trust by facilitating the intensifying demand for sensitive data for research purposes while ensuring privacy, confidentiality and safe access.^5–7^

Alongside the increasing prominence of TREs sits the drive for health data to feed into artificial intelligence (AI). AI is the prevailing umbrella term to refer to a range of computational techniques that can be used to make machines complete tasks in a way that would be considered intelligent were they to be completed by a human. Here we specifically refer to ML developments. This is a particular form of AI which involves computers ‘learning’ and adapting without specific instructions, doing so by using algorithms to analyse and draw inferences from data. With the UK’s healthcare sector being positioned as a unique data-rich ecosystem that could wield significant medical advances due to its centralised nature and longitudinal population data^8^ and as a lucrative business opportunity potentially worth several billions,^9^ there is a concerted push to realise the UK’s plans to become a global technological superpower^10^ via its national healthcare system. This increased interest in the application of ML on sensitive data (ie, special category personal dataiii), such as healthcare and medical data, means that TREs are increasingly approached with requests to use their data to develop new types of outputs, such as trained ML models. Such developments present new opportunities but also challenges for the secure and trusted function of TREs.

In order to advance these new opportunities for TREs while maintaining high privacy standards, the Data and Analytics Research Environments UK (DARE UK) Sprint Project titled Guidelines and Resources for AI Model Access from TrusTEd Research environments (GRAIMATTER) investigated the additional risk posed for the disclosure of personal data introduced by the release of trained ML models from TREs, and developed a set of technical, legal and ethical recommendations for how TREs should carry out disclosure control on ML models. Reflecting on our work as the Legal and Ethical project subteam, in this article, we focus on key ethical and legal issues stemming from the training of ML models from TRE data and how this impacts on TREs’ operation. While the export of research output from TREs is generally regulated through controls such as manual supervision (‘eyeballing’) to ensure no personal data leave the TRE, the situation changes when TRE exports present more complex configurations such as ML models which then may be released to open source repositories such as GitHub. In such cases, it becomes harder to identify potential risks using conventional manual checks and therefore harder to guarantee the ‘T’ of TREs. To address these points, we will (1) explain what a TRE is and how it works. In particular, we will discuss how the ‘Five Safes’ framework contributes to their use and governance. We will then (2) address the relationship between the digitisation of healthcare in the UK and data-intensive innovations such as AI, and specifically ML, while identifying how TREs are being positioned as a way to ensure much needed public trust in such developments. We will conclude (3) by highlighting three legal and ethical critical areas that require further consideration for TREs and others involved in ML healthcare research for public benefit. This is significant for TREs, and for health data research more broadly, as ML research may disturb the current balance struck between facilitating research and protecting privacy in TREs given the risk of disclosure of sensitive personal data once a trained ML model is exported from the TRE, and the lack of clarity in terms of legal responsibility were a data breach to occur. This also relates to the dynamic nature of models compared with the ‘static’ nature of traditional TRE outputs, which may also require a more dynamic ethics process accompanying the research, and in turn require a rethinking of the public benefit produced by such research.

## What is a TRE

A TRE is a secure physical or virtual environment designed for approved and named researchers to access sensitive pseudonymised data, where access to specific data sets is provided only to approved research projects. TREs differ from other data use models such as the more traditional *data release model*, where data are made available to approved researchers to download and analyse in their own data environments, hence risking losing control of their security and management. Instead, in a TRE, data are not released externally to data users for analysis on their own computers but placed on a server within a restricted, secure information technology environment, where the approved user is given secure access to carry out their project analysis. No row-level data leave the TRE environment. Traditionally, only aggregate-level results (eg, summary tables, graphs, statistical models) are released from a TRE at the end of the project, and only after a range of automatic and manual screening controls are applied to ensure that all outputs are non-disclosive of personal data.

The use of TREs is meant to address the challenges of using health and other forms of sensitive personal data to facilitate research that is assessed to be in the public interest while at the same time protecting privacy and ensuring trustworthiness.^5 6^ Importantly, their use does not eliminate the risk of disclosure of sensitive personal data but greatly mitigates it^11^ by providing assurances that data are handled securely, as data use can be tracked and technical and organisational measures are in place to check that no data leave the secure environment. Acting as data processors, TREs are meant to maintain a balance between:

- Confidence of data controllers (who determine the purposes for and manner in which any special category personal data are to be processed) through increased security.
- Benefits to the user/researcher (who can be from an academic, commercial or government setting) through improved access to larger data sets.
- Transparency for public and patients (whose personal data are made available in pseudonymised form) as to who has access to the data and for what purposes in order to ensure their continuing confidence and engagement.^7^

Robust data governance is key in achieving and maintaining such a balance. This means meeting all relevant legal obligations (eg, data protection, confidentiality, contracts and intellectual property), technical and cybersecurity requirements, and research ethics and data governance requirements.

There are several related frameworks used for providing guidance on TRE governance^iv^ most of which are based on the ‘Five Safes’ model. ‘Five Safes’ is an internationally recognised model introduced by the UK Office for National Statistics in 2003. It has been described as an ‘explicitly relativistic, subjective and empirical’ framework which has proved a ‘useful’ tool to frame, rather than prescribe, the crucial discussions around governance and management of sensitive data involving data providers, users and regulators.^12^ The ‘Five Safes’ breaks down the decisions surrounding data access and use into five related but separate dimensions:^13^ ^v^

### Safe people

TRE staff and the researchers accessing the data through a TRE are trained and authorised to use the data safely, follow guidelines and report data safety concerns, if any.

### Safe projects

Through an initial ethical and data governance approval process, TREs ensure that the research projects are approved by data controllers, and that data are used appropriately and for public benefit.

### Safe outputs

TREs screen all outputs thoroughly and approve the release only after ensuring that it does not include personal data.

### Safe data

The data are deidentified/pseudonymised before access is granted to researchers. It is ensured that researchers only see the data that they need to.

### Safe setting

TREs provide a safe environment to access personal data and prevent any unauthorised use.

## AI and the digitisation of healthcare: improving safety through the use of TREs

Healthcare has been identified as ‘one of the most important sectors for AI both for better services and for better efficiency’.^14 15^ This has paved the way for new, and arguably controversial, public–private–academic partnerships^16^ for the development of new AI technologies, including ML, which can be used in several healthcare areas such as diagnostics, therapeutics, population health management and administration, and for providing key infrastructure for the storage, maintenance and management of the data that underpin these technologies.^17^

Such developments align with the ongoing efforts since 2002 towards the digitisation of the NHS—from (missed) aims of achieving a ‘paperless’ NHS by 2018 to the renewed target for a ‘core level of digitisation’ by 2024^18^—which has resulted in a rich and valuable wealth of healthcare data. The COVID-19 pandemic has reconfirmed and further accelerated plans for the digitisation of the NHS (ie, NHS apps, virtual appointments, online treatments, etc), along with recent plans to facilitate the more effective sharing of digital health and social care records and data.^19^ Further plans to personalise healthcare through the use of wearable technologies and apps will only enrich these data sets.

AI, and in particular ML, technologies for healthcare rely on the availability of big data for their training and development. As such, the extensive medical and healthcare data that result from the ongoing interactions between the UK public and the NHS have long been seen as a prime opportunity for the adoption of innovative AI technologies, for day-to-day patient care and for the further advancement of health research.

While the opportunities that the increasing digitisation of healthcare offers appear exciting, the risks and concerns that such developments entail are considerable. There have been a series of situations where public trust has been eroded in data sharing—some of which have attracted significant media attention and regulatory enforcement, while others may be more ‘routine’ infractions of contracts. Nevertheless, these instances cumulatively may instil a negative attitude in the public towards data sharing. Among these, past big data health initiatives, such as care.data—an English initiative designed to allow the repurposing of primary care medical data for research and other purposes—and more recently the postponed GP Data for Planning and Research programme,^20^ demonstrate the importance of public trust for major projects which seek to aggregate and centralise healthcare and related data, and the costly danger of losing it if legitimate public concerns are not taken seriously.^21 22^ Scandals, such as the ongoing case of DeepMind/Google and the Royal Free,^vi^ along with a recent report in the *BMJ* that there are hundreds of organisations such as clinical commissioning groups, private companies and universities which have breached patient sharing agreements, some of them with little or no consequences^23^ (see also ref 24 25), demonstrate that what is often termed the ‘deficit of public trust’^26^ is not the result of public ignorance or badly publicised information^21 27^ (see also ref 28 29). Instead, it is an appropriate response of a public who, while willing for their data to be used for the benefit of patients and the NHS, are wary of a weak regulatory landscape that allows such data security failures.^2 30^ Nevertheless, this emerging picture has received limited attention in the academic literature and limited discussion as to how it may impact on data sharing and governance arrangements and policies more broadly as the NHS seeks to digitise and share more data.vii

To address the issue of the protection of personal data and the facilitation of research, TREs are positioned as a way to maintain and restore public trust.^5–7^ The technical and organisational safety measures that TREs offer can provide assurances that, not only will data not be leaving their secure environments but every interaction and subsequent analysis will be checked and tracked. Furthermore, their commitment to *Safe Projects* means that each project is assessed by an ethical and data governance committee for their potential to public benefit before a project approval is granted.viii In order to ensure public benefit and build public trust, the importance of patient and public participation alongside transparency of decision-making and data use has been further highlighted.^7 31 32^

However, while TREs may be identified as the appropriate way to address public trust concerns, our research shows that the increase in the development and adoption of AI, and in particular ML, in the medical and healthcare fields presents new challenges for the next generation of TREs which may threaten the ‘T’ of the TREs due to additional risks of disclosure of personal data by ML models trained on TRE data and a lack of clarity about chains of responsibility once the ML model has left the TRE environment.

## TRE outputs and ML

Typically, TRE outputs take the form of aggregated results, graphs and tables. Before their release, these outputs go through both automatically and manually disclosive controls and are checked to ensure that no identifiable information is attached to them before allowed to leave the TRE. With the increased interest in ML trained on special category data such as healthcare and medical data, TREs are increasingly approached with requests to use their data for such purposes and to disclose new types of outputs, such as ML models trained on TRE data.

As the GRAIMATTER research demonstrates, the release of trained ML models from TREs introduces an additional risk for the disclosure of personal data.^13 33^ In other words, while on the one hand models are being constructed using training data, on the other, training data and/or a semblance or subset of it, or information about who was in the training set can also, in certain cases, be reconstructed from a model. This means that trained ML models may be considered as containing personal data and therefore constitute personal data sets, bringing them within the jurisdiction of data protection legislation.^34^ Personal data disclosure from trained ML models can happen inadvertently—for example, if the ML algorithm is overtrained, and the weights of the algorithm which are then exported from the TRE correspond to the data underneath—or, there can be malicious intent—for example, when a malicious researcher ‘hides’ individual-level data within the files (eg, sensitive data could be embedded in the weights of an ML algorithm which are then exported).^33^ ^ix^

In order to mitigate any risk of direct or indirect personal data breach from the disclosure of trained ML models, and hence maintain public trust, key aspects of the technical, legal and ethical governance of TREs need to be reconsidered. Our project GRAIMATTER explored these challenges and proposed a range of measures and recommendations that need to be considered for the safe disclosure of trained ML models from TREs.^13^ We focused specifically on the ethical and legal governance issues arising from such practices recommending ways that they can be addressed. Here we present some broader issues that informed our thinking.

## Legal and ethical challenges

While TREs have been positioned as the safer response to the riskier and controversial data release model, it is important to highlight that they are not a magic bullet. All their technical controls notwithstanding, they too are complex sociotechnical systems which, each in their own ways, bring together people, technology, regulations, institutional bodies, auditing and organisational procedures in a sophisticated but always precarious balance that seeks to facilitate research access to data while preserving privacy. The introduction of ML in such a setting requires us to rethink carefully whether and how a new balance can be achieved. In the paragraphs that follow, we highlight three critical areas that require further consideration if we want to ensure a ‘safe’ health and care data environment that truly serves the public, namely the possibility of disclosure of personal data by ML models once they have left the TRE, the dynamic nature of ML models and the impact that these and other factors have on the discussions around public benefit.

### Disclosure of personal data in TREs

While TREs only make available pseudonymised data for research purposes, pseudonymisation is a risky process that can lead to reidentification when combined with additional information. This is a known risk that can be mitigated by contractual agreements between TREs and researchers within the legal framework of the Data Protection Act 2018 which covers data that, if processed, could lead to reidentification within the definition of ‘personal data’. As per section 171 of the Data Protection Act 2018, fines and criminal penalties are meant to act as a deterrent to any researcher who would attempt to reidentify them.^x^

The case of ML models within TREs complicates matters. As our GRAIMATTER project team has demonstrated, there is indeed a risk that an ML model leaving the TRE could be disclosing data that could lead to reidentification and therefore constitute personal data.^13^ However, it is debatable whether the ML model, per se, could be classified as ‘personal data’, and hence fall under the data protection framework or not.^34 35^ If it is personal data, there is a legal responsibility that both the risks of the specific projects and the controls taken to mitigate them should be clearly specified before the release of the model. Currently, there is a lack of guidance from the Information Commissioner’s Office on what form these controls should take, and on whom this responsibility falls. While these issues could be addressed by drawing new contractual agreements, or updating existing ones between the data controllers and the researcher, it is important for the relevant regulatory body to provide clear and updated guidance on a national level to address such risks.^xi^

### Dynamic nature of ML models

The dynamic nature of ML models means that their life does not come to an end after one application. After the model leaves the TRE it might move between different applications and uses. Its interaction with different data sets might render data identifiable further down the line. However, by then, the chain of legal responsibility may be unclear and existing legal frameworks do not yet provide sufficient guidance on how the changing and dynamic nature of ML algorithms and models can be regulated.xii

Besides legal issues, the dynamic nature of these models also raises ethical concerns as it makes it difficult to identify and assess the risks that the TRE export of ML models may entail. Typically, the ethical assessment process conducted by TREs relies on assessing the benefits but also potential risks of the proposed project before judging whether approval should be granted. The element of unpredictability introduced by the disclosure of ML models risks undermining this process as it is impossible to determine whether future interactions of the ML model with different data sets after its TRE release might introduce new risks or what their level might be. Therefore, the traditional ethical process governing research using TREs needs to be rethought and new strategies must be developed that can respond to the challenges that ML models pose.^36^ For example, instead of limiting the ethical assessment process to the application stage, a more dynamic approach whereby multiple ethical checks are conducted before and after the project is developed, and as an ML model is released, might prove more appropriate.^37^ Should these regular checks reveal a modification to the risk of data breach, a new overall ethical assessment should be undertaken in order to minimise future damage.xiii

### Rethinking public benefit

The concept of public benefit has been identified as the ‘critical safeguard’ for the safe and appropriate use of health and care data,^31^ and in TREs it is key for the delivery of *Safe Projects*. However, if we are to take this concept and our commitment to it seriously, we need to calibrate the public debate to more accurately reflect the risks, difficulties, unknowns and harms that surround data-intensive healthcare research, especially involving AI and ML, along with the asymmetrical ways that these are distributed.

Indeed, despite early warnings of the hype that surrounds AI in healthcare (and beyond), it is not often we hear about the unpredictable ways that AI healthcare technologies can fail.^38^ Or about the scarcity of actual clinical trials to prove the safety, the clinical potential or the efficacy of AI medical tools.^39 40^ Beyond the excitement, there is little expert knowledge on how operational changes or changes in the diversity and volume of data can impact on the performance of AI algorithms that are already in use with the potential of seriously undermining patient safety,^41^ or few public conversations about the trade-off between AI efficacy and data privacy (ie, the more accurate an AI algorithm, the less private it is). A recent policy report warned that ‘attention-grabbing‘ AI technologies can sometimes ’crowd-out‘, in terms of funding, other conventional but still essential work in a chronically underfunded NHS,^6^ while others warn that algorithms are already creating and worsening health inequities.^42 43^ There is scarce discussion that the intense computational processes that AI and big data technologies rely on have a big, and unevenly distributed, environmental impact that needs to be factored in,^44^ or that the ‘essential infrastructures’^45^ that they require are to be delivered by the private corporations that the public has repeatedly warned against.

As transparency is central in ensuring public benefit and building public trust, we need an honest, grounded and sophisticated public discussion about what is the public benefit that underwrites these AI and ML developments, how and by whom it is being assessed and at what and whose cost. While this should not mean ignoring the benefits that such innovations could bring forth, it may mean that current ways of assessing and ensuring public benefit in TRE operation and use of TRE data need to be rethought in light of the potential benefits but also broader challenges of AI development and use.

## Conclusion

In this article, we have highlighted developments in health data and ML research and policy vis-a-vis TREs in the UK. We identified how TREs are being positioned at the junction between an increasing appetite for data-driven and ML healthcare technologies and an increasing awareness of the importance of legal and ethical safeguards guiding such innovations. Drawing from our work on the GRAIMATTER project which explored the additional risks when disclosing trained ML models from TREs,^13^ we first explained what TREs are and how the ‘Five Safes’ framework contributes to their use and governance. We then addressed the relationship between the digitisation of healthcare in the UK and data-intensive innovations such as AI, and in particular ML, while identifying how TREs are being positioned as a way to ensure much needed public trust in such developments. We concluded this article by highlighting three broad legal and ethical critical areas that require further consideration if we want to ensure a ‘safe’ health and care data environment that truly serves the public: (1) the risk of personal data being disclosed in ML models, (2) the dynamic nature of ML models and (3) how public benefit may be (re)conceived in this context. We argue that these broad critical areas require further thought from TREs and others involved in the UK health data policy ecosystem if they want to ensure a truly ‘safe’ health and care data environment that indeed serves the public while facilitating AI and ML research on UK health data.

## Data Availability

Data sharing not applicable as no data sets generated and/or analysed for this study. Not applicable.

## Footnotes

For example, in June 2022, the Department of Health and Social Care published its data strategy for health and care in England titled ‘Data Saves Lives: Reshaping Health and Social Care with Data’.^5^ In 2021, Scotland published its updated digital health and care strategy.^46^ In Europe, the European Commission is working on the eHealth programme.^47^

For example, the Secure Research Service run by the ONS (ONS SRS) has been operational since 2003 and has provided the blueprint for many subsequent TREs. In Scotland, a system of safe havens (four regional and one national safe haven) has been in operation for over a decade now. In Wales, the secure research platform SeRP was created to store data for the SAIL Databank that collects and manages all public sector data of Wales, since 2005.

As per Data Protection Act, 2018, section 10 (c).

For example, the five TREs in Scotland follow the Charter for Safe Havens in Scotland. The charter draws from *the Guiding Principles for Data Linkage* (which in turn draws on human rights legislation, the Data Protection Act, guidance from the Information Commissioner and the Scottish Government Identity Management and Privacy Principles), the *SHIP Blueprint* and associated governance frameworks that define standards and process for the use of non-consented linked data for health informatics research in Scotland. In 2021, the UK Health Data Research Alliance published a set of principles and best practices^7^ structured around the ‘Five Safes’ framework and further inspired by the OECD Guidelines on Human Biobanks and Genetic Research Databases, work of NHSX, NHS Digital, the National Data Guardian and through guidance from the Information Commissioner.

In some cases, the 'Five Safes' model has been extended to include ‘Safe Return’^48^ which has been coined the ‘Five Safes Plus One’ approach,^49^ and ‘Safe Computing’ as an extension of ‘Safe Setting’.^7^

Here we are referring to the now infamous case of the Royal Free releasing, in 2015, millions of their patient data to the AI company DeepMind (later acquired by Google) for the development of a medical app without the appropriate legal and ethical safeguards for their protection. In 2017, the UK’s ICO sanctioned the Royal Free for breach of UK data protection law. While back then Google avoided sanctions, it is currently being sued in a private litigation class action lawsuit for the unlawful use of patients’ confidential medical data^50–53^ (see also ref^54-56^).

The merging of NHS England and NHS Digital in February 2023 will provide ample scope for complicating a complex structure further, https://digital.nhs.uk/about-nhs-digital/nhs-digital-merger-with-nhs-england (accessed 1 Feb 2023).

The make-up of these committees differs between TREs while depending on the project’s design, methods and data needs further governance approvals might be deemed necessary (eg, NHS R&D, or Caldicott approvals).

Other threats which can result in the recovery or reconstruction of personal data, including special category personal data, after the ML model has left the TRE are membership inference attacks and model inversion attacks.^13 33^

Notably, public bodies especially in healthcare have rarely been the subject of fines from data protection regulators. The incoming UK Information Commissioner announced in June 2022 that fines would only be issued to public authorities ’in the most egregious cases’.^57^

The UK Government published a Data Protection and Digital Information Bill in June 2022, which represents its vision for reform of data protection law in the UK post-Brexit. With the change of prime ministers, this Bill has now been withdrawn, but it did include provisions relevant to research, which would have made it easier for researchers to use personal data for research purposes with an inverse effect on the privacy and data protection rights of the individuals whose data are being used.^58^ If the Government takes up data protection reform again, such reform needs instead to strengthen these rights and adequately address the risks posed by research especially vis-a-vis AI and ML.

Interventions such as the proposed AI Act in the European Union^59^ aim to address this issue but currently there is no similar legislation or legislative proposals in the UK.

Notably, adopting such a dynamic approach to ethics is likely to require significant reform of existing ethics processes and significantly more resources than at present.
